# Development of a core set of outcome measures for OAB treatment

**DOI:** 10.1007/s00192-017-3481-6

**Published:** 2017-09-25

**Authors:** Caroline Foust-Wright, Stephanie Wissig, Caleb Stowell, Elizabeth Olson, Anita Anderson, Jennifer Anger, Linda Cardozo, Nikki Cotterill, Elizabeth Ann Gormley, Philip Toozs-Hobson, John Heesakkers, Peter Herbison, Kate Moore, Jessica McKinney, Abraham Morse, Samantha Pulliam, George Szonyi, Adrian Wagg, Ian Milsom

**Affiliations:** 1 Department of Obstetrics and Gynecology, Division of Female Pelvic Medicine and Reconstructive Surgery, Massachusetts General Hospital, Boston, MA USA; 2 International Consortium for Health Outcomes Measurement, Cambridge, MA USA; 3 Department of Urologic Reconstruction, Urodynamics, and Female Urology, Department of Surgery, Division of Urology, Cedars-Sinai Medical Center, Los Angeles, CA USA; 4 Department of Urogynaecology, King’s College Hospital, London, UK; 5 Bristol Urological Institute, Southmead Hospital, Bristol, UK; 6 Section of Urology, Department of Surgery, Dartmouth-Hitchcock Medical Center, Lebanon, NH USA; 7 Birmingham Women’s NHS Foundation Trust, Birmingham, UK; 8 Department of Urology, Radboud University Medical Center, Nijmegen, The Netherlands; 9 Dunedin School of Medicine, University of Otago, Dunedin, New Zealand; 10 Department of Urogynaecology, University of New South Wales, Sydney, NSW Australia; 11 Center for Pelvic and Women’s Health, Marathon Physical Therapy and Sports Medicine, LLC, Norton, MA USA; 12 Department of Obstetrics and Gynecology, Guangzhou Women and Children’s Medical Center, Guangzhou, China; 13 Division of Urogynecology and Reconstructive Pelvic Surgery, Department of Obstetrics and Gynecology, University of North Carolina, Chapel Hill, NC USA; 14 Department of Geriatric Medicine, Royal Prince Alfred Hospital, Sydney, Australia; 15 Geriatric Medicine, University of Alberta, Edmonton, Canada; 16 Department of Obstetrics & Gynaecology, Institute of Clinical Sciences, Sahlgrenska Academy at Gothenburg University, Sahlgrenska University Hospital, SE-416 85 Gothenburg, Sweden

**Keywords:** Overactive bladder, Outcome measures, Standard set

## Abstract

**Introduction and hypothesis:**

Standardized measures enable the comparison of outcomes across providers and treatments giving valuable information for improving care quality and efficacy. The aim of this project was to define a minimum standard set of outcome measures and case-mix factors for evaluating the care of patients with overactive bladder (OAB).

**Methods:**

The International Consortium for Health Outcomes Measurement (ICHOM) convened an international working group (WG) of leading clinicians and patients to engage in a structured method for developing a core outcome set. Consensus was determined by a modified Delphi process, and discussions were supported by both literature review and patient input.

**Results:**

The standard set measures outcomes of care for adults seeking treatment for OAB, excluding residents of long-term care facilities. The WG focused on treatment outcomes identified as most important key outcome domains to patients: symptom burden and bother, physical functioning, emotional health, impact of symptoms and treatment on quality of life, and success of treatment. Demographic information and case-mix factors that may affect these outcomes were also included.

**Conclusions:**

The standardized outcome set for evaluating clinical care is appropriate for use by all health providers caring for patients with OAB, regardless of specialty or geographic location, and provides key data for quality improvement activities and research.

**Electronic supplementary material:**

The online version of this article (10.1007/s00192-017-3481-6) contains supplementary material, which is available to authorized users.

## Introduction

Overactive bladder (OAB) is a symptom-based condition defined as urinary urgency, with or without urgency incontinence, usually accompanied by frequency and nocturia in the absence of urinary tract infections or other obvious pathology [1, 2]. It is a common condition that affects many individuals worldwide, with a prevalence estimated between 11.8 and 17%, with incidence increasing with increasing age. OAB negatively impacts quality of life (QoL) and often results in significant healthcare expenditures [3–5]. Treatments range from conservative approaches, such as lifestyle intervention, to pharmacological and surgical options. For many patients, the process of seeking care involves frequent clinic appointments and multiple treatment approaches. A study of five European countries and Canada found that the annual expenditure per patient for OAB ranged from 262 to 619 euros (US$293–693). When indirect costs such as work absenteeism were included, the total cost for the estimated 25 million people with OAB in the countries studied was 9.7 billion euros [6]. Despite the significant cost of OAB management, it is difficult to determine the most effective and efficient treatment approaches because there are no standard outcome metrics that allow comparison of outcomes and costs across providers.

The discipline of value-based health care (VBHC) contains the tools to support such a strategy [7]. VBHC defines “value” in healthcare as the ratio between the outcomes of care delivered and the cost of achieving those outcomes. The VBHC agenda focuses on the standardization of metrics to promote comparison of outcomes and costs across providers for identifying best practices for delivering high-value care [8]. Standardized measures enable comparison of outcomes across providers and treatments to improve care quality and efficacy. Measures for evaluating care outcomes for OAB that are most important to patients would be of great utility to improve our understanding of which treatment options or combinations offer greatest treatment value.

The International Consortium for Health Outcomes Measurement (ICHOM) is a not-for-profit organization that convenes international working groups of clinicians, researchers, and patients to define minimum sets of standardized outcomes by medical condition with a focus on outcomes that matter most to patients (www.ichom.org). Following such development, ICHOM works to support implementation and benchmarking of these standard sets to facilitate the adoption of VBHC worldwide. The objective of this project was to define a minimum standard set of outcomes for evaluating OAB treatment. This set of outcome measures and case-mix factors is designed to be appropriate for and easily implemented by any clinician treating patients with OAB, regardless of medical specialty, treatment given, or country of practice.

## Methods

### Working group

ICHOM convened an international working group (WG) of clinicians, researchers, and patients who are experts in treating OAB. Members were selected via review of the literature and consultation with leaders in the field to provide global representation across the key clinical disciplines involved, including urology, urogynecology, geriatrics, and pelvic floor physical therapy (Table 1). The WG was led by a project team composed of an ICHOM standardization director (SW), co-leads (AW and IM), and research fellow (CF). All members were physicians, three routinely treated patients with OAB (AW, IM, CF), and all volunteered their time for the project. The standardization director and research fellow were employed by ICHOM. Funding for the project was provided by the International Urogynecology Association (IUGA). To better understand which outcome domains matter most to patients, the project team invited a patient representative to the WG and held patient-group discussions.

**Table 1:** Working group members by country and specialty, including organizations and specialty societies represented

Country	Specialty	Working group member	Organization	Specialty society
Australia	Geriatric medicine	George Szonyi	Royal Prince Alfred Hospital	CFA
		Kate Moore	School of Women’s and Children’s Health, University of New South Wales	ICS, CFA
Canada	Geriatric medicine	Adrian Wagg	University of Alberta	ICS ICI
The Netherlands	Urology	John Heesakkers	Radboud University Medical Center	EAU (AUA, ICS, SUFU)
New Zealand	Biostatistics	Peter Herbison	Dunedin School of Medicine, Otago University	Cochrane
Sweden	Obstetrics and gynecology	Ian Milsom	Sahlgrenska Academy	EUGA, IUGA, ICS ICI
United States	Obstetrics and gynecology	Caroline Foust-Wright	Massachusetts General Hospital	
	Patient representative	Anita Anderson		
	Physical therapy	Jessica McKinney	Marathon Physical Therapy & Sports Medicine, LLC.	
	Urology	Jennifer Anger	University of California - Los Angeles	SUFU
	Urogynaecology	Elizabeth Ann Gormley	Dartmouth-Hitchcock Medical Center	AUA, SUFU
		Abraham Morse	Guangzhou Women and Children’s Medical Center	AUGS
		Samantha Pulliam	University of North Carolina at Chapel Hill	AUGS
United Kingdom	Pelvic floor medicine	Philip Toozs-Hobson	Birmingham Women’s NHS Foundation Trust	IUGA
	Outcomes research	Nikki Cotterill	Bristol Urological Institute	ICI
	Urogynaecology	Linda Cardozo	King’s College Hospital, National Health Service (NHS)	EUGA (IUGA), BSUG, ICS, ICI

*CFA* Continence Foundation of Australia,* ICS* International Continence Society,* EAU* European Association of Urology,* AUA* American Urological Association,* EUGA* European Urogynaecology Assocation,* IUGA* International Urogynecological Association,* SUFU* Society of Urodynamics, Female Pelvic Medicine & Urogenital Reconstruction,* AUGS* American Urogynecologic Society,* ICI* International Consultation on Incontinence,* BSUG* British Society of Urogynaecology

### Standard set scope

The WG decided that the standard set would apply to outcomes of care for all adult patients diagnosed with idiopathic OAB, excluding residents of long-term care facilities, and unanimously agreed to define OAB according to the International Continence Society (ICS)/IUGA definition. Treatment approaches included first-line interventions such as patient education and behavioral modification, bladder retraining, pharmacological management, onabotulinumtoxinA injection, posteroposterior percutaneous tibial nerve stimulation (PTNS), sacral neuromodulation (SNS), and surgery.

### Work process and decision making

The measure set was developed using a modified Delphi process [9]. Between September 2015 and June 2016, the group convened for eight teleconferences. Each addressed a specific goal: establishing the scope of the measure set, defining the patient population, selecting the appropriate outcomes and case-mix domains, and defining the relevant metrics. For each topic, the project team reviewed the existing literature and current practices to develop proposals for discussion during the teleconference. Detailed minutes of these discussions were distributed to WG members, who then voted on each item presented in the proposals via an online survey. Individual proposal items required a 70% majority vote of survey respondents to be included in the measure set. Survey items with < 70% approval were either excluded from the set or revised by the project team following comments and were again presented for discussion and voting at the next teleconference.

### Selection of outcome and case-mix domains

Additional sources of information were sought to support the selection of outcome domains. A systematic literature review was performed to determine outcome domains currently used to evaluate OAB. PubMed was searched with the terms “overactive bladder” or “urinary bladder, overactive” or “lower urinary tract” combined with “patient outcome assessment” or “outcome assessment” or “treatment outcome(s)”. Limits included full-text articles, humans, publication from 1 January /2000 to 31 December 2015, English language (due to the language limits of the team leadership), and data from earlier publications in the form of review articles and meta-analyses (complete description provided in Supplementary Material 1).

Structured patient group discussions were conducted to better understand what outcome domains mattered most to patients. We aimed to balance this group by age, parity, phase in the care cycle, clinical experience, and nationality. We recognize that this did not provide a fully representative sample of patients globally, however, our aim was to gather information to guide our work, not to publish definitive results. Specifically, patient groups were asked which outcomes were most important to them, had the most effect on day-to-day life, and if any domains were missing from those identified from the literature review.

A comprehensive list of potential outcome domains was identified from these sources and presented to the WG for discussion. WG members were asked to score each potential outcome on the Grades of Recommendation Assessment, Development and Evaluation (GRADE) scale [10]. Outcome domains scored as important (7–9) by at least 70% of respondents were included in the set. Those scored as unimportant (1–3) by at least 70% of respondents were excluded. Those remaining were modified and represented for a second round of voting. Domains meeting neither inclusion nor exclusion criteria after a second round of voting were discussed again and then presented for a final binary vote.

Once outcome domains were identified, each was defined. All included domains were deemed appropriate for capture by patient report. Relevant patient-reported outcome measures (PROMs) were then identified from the literature and reviewed for coverage, psychometric properties, validity, feasibility to implement, and clinical interpretability, according to International Society for Quality of Life Research (ISOQOL) guidelines [11]. All PROMs proposed for discussion were denoted as Grade A by the 5th International Consultation on Incontinence (ICI) [12]. PROM sets that covered all outcome domains of interest while minimizing question burden were then presented to the WG for discussion and voting. A similar process was followed to identify and define case-mix variables, and patient and procedural factors known to affect treatment outcomes for inclusion in the standard set.

### Patient validation surveys

To ensure robust patient input, we solicited feedback via an anonymous online survey publicized via national continence-related consumer organizations. The survey presented in lay terms outcome domains voted for inclusion by the WG. Respondents were asked to score those domains according to their importance on the GRADE scale and given an opportunity at the end of the survey to suggest any missing outcomes. The resulting suggestions were presented to the WG to inform their conclusions on the generalizability of the patient advisory group.

### Open review process

To ensure transparency in the development process and allow input from stakeholders outside the formal WG, a 4-week open review period was held prior to the last WG teleconference. Key stakeholders identified by the project team, IUGA members, and individuals expressing interest in the measure set via the ICHOM website received an overview of the set, with links to the full detail reference guide and a feedback survey. Results of this survey were presented to the WG for discussion prior to set finalization.

## Results

### Outcome domains and measures

The literature search for outcome domains (Supplemental Material 1) identified 585 articles. An additional 11 articles were identified from other sources during the process. A title and/or abstract review was used for further refinement. A total of 184 articles were included for full-text review, and 39 were included in the final qualitative synthesis. After reviewing results of the literature search and patient discussion groups, the WG voted to include the following outcome domains in the standard set: symptom frequency and burden, physical functioning, interference with desired activities, emotional health, social interactions, sexual functioning, treatment burden, and overall satisfaction with the results of treatment. See Table 2 for a complete list of outcomes and outcome measures.

**Table 2:** Outcome domains and assessments in the standard set

Category and outcome domain	Agreement^a^	Outcome assessment	Agreement^a^
OAB symptom severity and burden			
Frequency of OAB symptoms	92	Tracked via ICIQ-OAB	83
Burden of OAB symptoms	100	Tracked via ICIQ-OAB	83
Health-related quality of life			
Physical functioning	100	Assessed via the OAB-Q SF	85
Social impact	88	Assessed via the OAB-Q SF	85
Emotional health	100	Assessed via the OAB-Q SF	85
Interference with desired activities	75	Assessed via the OAB-Q SF	85
Sexual functioning	73	Assessed via the ICIQ-FLUTSsex (women) or ICIQ-MLUTSsex (men)	83
Treatment benefit and tolerance	82	My condition (urinary problems, incontinence) has…(greatly improved/improved/not changed/worsened during treatment). The tolerability of my treatment for OAB is…(inadequate, moderate, good, excellent).	77
Overall satisfaction with treatment	100	My overall satisfaction is…(extremely satisfied/very satisfied/satisfied/not satisfied with the treatment).	77

*ICIQ* International Consultation on Incontinence Questionnaire,* OAB* overactive bladder,* OAB-Q SF * symptom bother and health-related quality of life (HRQL) questionnaire,* FLUTS* Female Sexual Matters associated with Lower Urinary Tract Symptoms, *MLUTSsex* Male Sexual Matters associated with Lower Urinary Tract Symptoms

^a^Percentage agreement among survey respondents (voting IN)

The International Consultation on Incontinence Questionnaire Overactive Bladder Module (ICIQ-OAB) is an eight-question instrument assessing the frequency of micturition, nocturia, urgency, and incontinence and the amount of bother caused by these symptoms [13, 14]. This PROM is validated for assessing OAB, easy to complete, and free for use in clinical practice and routine outcome measurement and was therefore recommended by the WG.

The OAB-q, 26 questions of which form the ICIQ-OAB-q, is a well-validated and commonly used instrument to assess the effect of OAB on health-related QoL covering the domains of coping, concern, sleep, and social interactions. The WG felt that these domains mapped closely to the domains of physical functioning, interference with desired activities, emotional health, and social interactions that had been voted for inclusion in the standard set [15]. However, at 33 questions long, it was considered by the WG to be too lengthy for use in standard clinical practice. Instead, the 13-item short form of the OAB-Q (OAB-Q SF) was recommended [16].

The WG voted to measure sexual function with the six-item ICIQ Femala/Male Sexual Matters associated with Lower Urinary Tract Symptoms (FLUTS/MLUTSsex) as applicable according to gender [14, 17]. The Treatment Benefit Scale (TBS), consisting of three questions assessing treatment efficacy, treatment tolerability, and overall satisfaction with treatment, was included to assess the domains of treatment benefit, burden, and satisfaction [18].

The resulting ICHOM standard set on OAB comprises 28 items.

### Patient validation surveys

Patient validation surveys revealed strong support for outcomes selected by the WG. A total of 99 complete responses to the survey was received from patients spanning a wide range of ages and treatment approaches; 76% of participants responded affirmatively to the question: “Do you feel this list captures the outcomes that matter or have mattered the most to you?” No major deficits in domain coverage were identified.

### Case-mix factors

Demographic and medical history data that might affect patient treatment outcomes were included in the standard set to allow for risk-adjusted outcomes comparisons. See Table 3 for a complete list of the case-mix factors and definitions.

**Table 3:** Case-mix variable domains and definitions included in the standard set

Category and case-mix factor domain	Agreement^a^	Case-mix factor definition	Agreement^a^
Demographic factors			
Age	100	Year of birth	93
Sex	100	Patient sex	86
Baseline clinical factors			
BMI	85	How much do you weigh? (Weight in kgs or lbs). How tall are you? (Height in cm or inches).	100
Comorbid bowel condition	71	Have you been told by your doctor or care provider that you have any of the following? Tick all that apply. 0 = None, 2 = Irritable bowel syndrome, 3 = Inflammatory bower disease (Crohn’s, ulcerative colitis).	86
Diabetes	77	Have you been told by your doctor or care provider that you have any of the following? Tick all that apply. 1 = Diabetes.	86
Cognitive impairment	86	Have you been told by your doctor or care provider that you have any of the following? Tick all that apply. 4 = A problem with your memory.	86
Coexisting pelvic organ prolapse	77	Do you have a feeling of a lump or “something coming down” or the need to manually replace a prolapse in order to empty your bladder?	93
Coexisting stress incontinence	71	Do you leak urine with physical activity, coughing, laughing, or sneezing or have you been told by a doctor that you have stress incontinence?	92
BPH or prostatitis	93	Have you been told by a doctor that you have a problem with your prostate? Tick all that apply. 0 = No, 1 = Enlarged prostate or benign prostatic hyperplasia (BHP), 3 = Prostatitis.	85
Current use of estrogens	92	Are you currently taking estrogens or hormone replacement therapy by mouth, a patch or cream on the skin, or as a suppository?	92
History of pelvic surgery	92	Have you had surgery to your pelvis? Please indicate what kind. Female response options: 0 = No; 1 = Yes, surgery for stress urinary incontinence; 2 = Yes, prolapse surgery; 3 = Yes, surgery to the rectum or bowel; 4 = Yes, hysterectomy; 5 = Yes, other surgery. Male response options: 0 = No; 1 = Yes, surgery to the rectum or bowel; 2 = Yes, prostate surgery.	92
Current OAB treatments	100	What are you currently using to treat your OAB symptoms?	100

*BMI* body mass index,* BPH* benign prostatic hyperplasia,* OAB* overactive bladder

^a^Percentage agreement among survey respondents (voting IN)

The WG voted to include only age and sex as demographic factors affecting OAB outcomes. Medications may have greater efficacy in patients < 65 years of age [19–25], and women respond better to treatment than do men [21, 22, 26, 27].

Aspects of patients’ medical history that may affect treatment outcomes or response to treatment included in the standard set were: body mass index (BMI), comorbid bowel conditions, pelvic organ prolapse (POP), enlarged prostate, history of prior pelvic surgeries, and diabetes or the metabolic syndrome. Women with higher BMI may be more likely to have urgency incontinence and increased symptom severity [28, 29]. Studies suggest a link between OAB and irritable bowel syndrome or other bowel conditions, although how this affects treatment outcomes is less clear [30, 31]. The association of OAB with anterior compartment prolapse resulted in the consensus to include POP [32]. As prostate conditions can increase storage symptoms, mimicking or worsening OAB and making treatment less successful, this condition was voted for inclusion in the case-mix factors [33, 34]. Histories of prior pelvic surgery were included due to reports of new-onset OAB following pelvic surgery [35]. Diabetes may exacerbate OAB, which was also included [36–40]. The presence or absence of memory problems was also included; the WG felt that, on balance, cognitive impairment could alter outcomes despite the lack of supporting published evidence. Including a measure that asks patients if they have been told by a doctor that they have memory problems will allow further investigation into this issue.

Parity, mode of childbirth (vaginal versus Cesarean), and menopausal status were ultimately excluded due to lack of clear data on their relationship to OAB symptoms [41–50]. Current use of estrogens was included, as topical estrogen has been shown to ameliorate OAB symptoms and systemic estrogen to be associated with worsening [51, 52].

Current OAB treatment is included in the standard set as an explanatory variable. By tracking patients’ treatment in parallel with their outcomes and medical profiles will help identify particularly effective treatments for different patient populations.

The WG opted to collect all case-mix factors via patient reports to simplify implementation of the standard set. As all outcomes in the set are patient reported, all data are captured from a single source, streamlining data collection within the clinic and eliminating the need to integrate PROM data collection in electronic medical records (EMRs) or to align patient-reported and clinical data following collection.

### Initial data collection and follow up

**Fig. 1:**
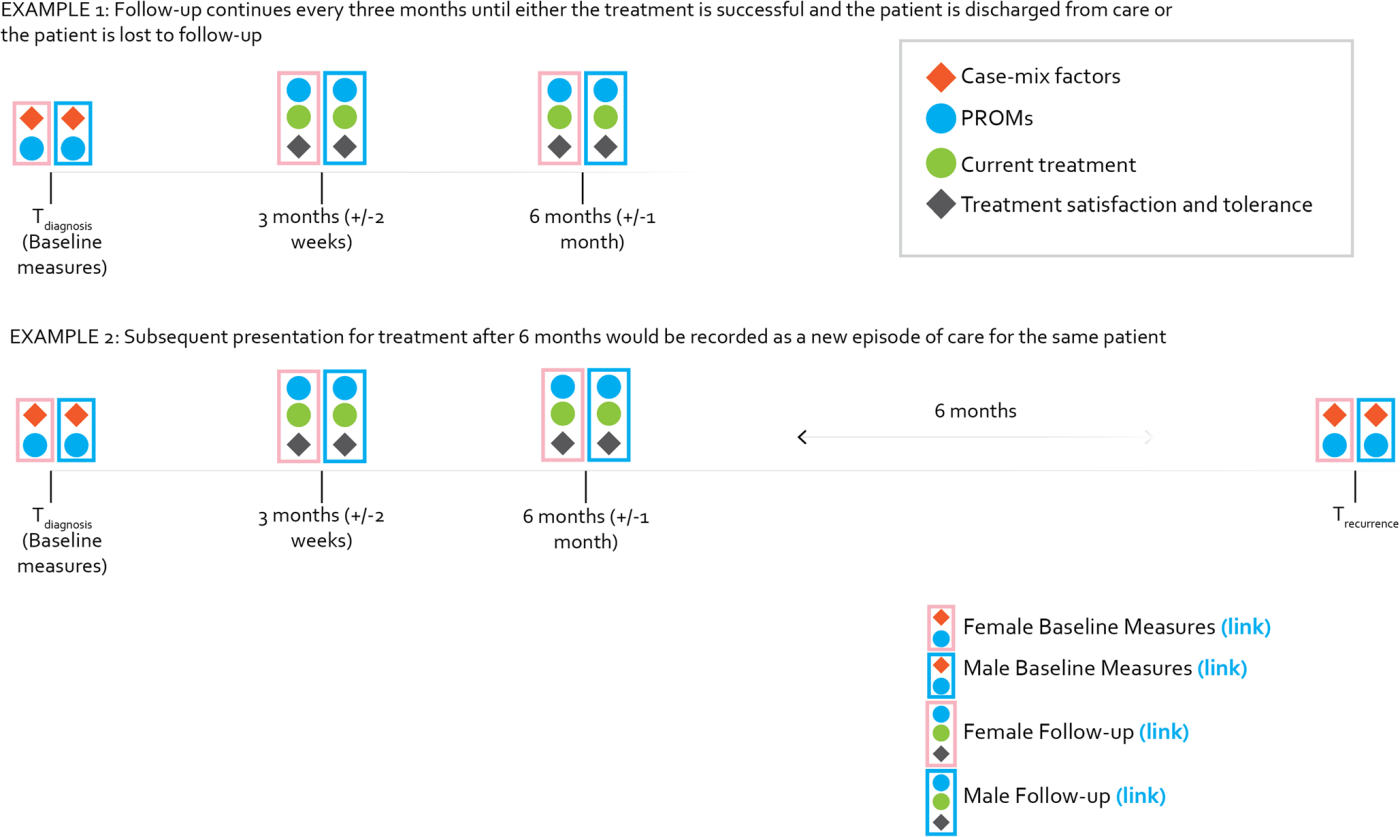
Follow-Up timeline and sample questionnaires. The* timeline* illustrates when standard set variables should be collected from patients, clinicians, and administrative sources. Links to the* sample questionnaires* may be found in the legend below

The standard set is designed for data collection to begin at the time of diagnosis, with follow-up at intervals defined by the treating clinician and to end at the time of patient-reported treatment success or loss to follow-up (Fig. 1). Baseline measures include both case-mix factors and all PROMs. Although studies suggest that baseline severity does not affect response to treatment, it may affect patient motivation to adhere to treatment and tolerate side effects [53, 54]. All PROMs are collected at the time of diagnosis to calculate the effect of treatment over time.

Follow-up measures include all PROMs and explanatory variables. The WG recommended that follow-up surveys be completed as deemed appropriate by the treating clinician. Follow-up ends at patient-defined success, i.e., positive responses to the TBS questions and/or no further follow-up for 6 months. Subsequent presentations for treatment after 6 months are recorded as new episodes of care for the same patient. Treatment outcome is then defined as the difference in PROMs at patient-reported treatment success or the last available follow-up survey and baseline adjusted by relevant case-mix factors.

## Discussion

The ICHOM OAB WG aimed to develop a comprehensive, minimally burdensome, patient-centered, standard set of outcome measures for evaluating the care of patients with OAB for use in routine clinical care. This led to the development of an outcome set that is efficient to collect and provides clinicians with a holistic view of treatment outcomes. This was accomplished by convening and guiding a WG of experts representing a broad range of stakeholders through a structured process, grounded in literature and expert opinion, to achieve consensus. Patients were included in the process to better determine what outcome domains mattered most to them. In addition, the WG limited its recommendation to measures that are free for use and broadly translated, allowing for set’s adoption around the globe. All variables in the set are collected by patient report, and follow-up intervals are left to the clinician’s discretion, as protocols may differ across specialties/countries. This allows for flexible data collection from the patient upon arrival at the clinic or remotely via mailed survey, patient portal, secure email, or app. It is important to note that the patient-reported case-mix factors were developed by the WG and therefore require further validation. To facilitate this validation and pilot the standard set as a whole, ICHOM supports members of the WG and other interested parties to implement the set and design validation studies. This work is overseen by a six-member steering committee, elected from the initial WG, that governs changes to the standard set over time.

The standard set was developed to have the lowest possible burden on patients and providers while collecting necessary data for comparison of outcomes. Thus, some measures discussed by the WG were not included in the final set: for example, a bladder diary (voiding diary) was not included to decrease the burden on patients of data collection. It was felt that tracking changes in symptom frequency and burden via the ICIQ-OAB adequately captured symptom severity and frequency.

In conclusion, this standard measure set provides meaningful, comparable, and easy-to-interpret measures for evaluating the care of patients with OAB. The inclusion of case-mix factors enables global comparisons of treatment outcomes across population groups. In time, it is anticipated/proposed that knowledge from these comparisons will encourage and empower providers to improve care and allow patients, providers, and payers to make informed decisions about their healthcare spending and treatment options.

## Electronic supplementary material

ESM 1 (DOCX 116 kb)

## Abbreviations

OAB: Overactive bladder
ICHOM: International Consortium for Health Outcomes Measurement
WG: Working group
VBHC: Value-based health care
IUGA: International Urogynecology Association
ICS: International Continence Society
PTNS: Posteroposterior percutaneous tibial nerve stimulation
SNS: Sacral neuromodulation
