# Supplementary material for: Development of a core set of outcome measures for OAB treatment
Source: Int Urogynecol J. 2017 Sep 25;28(12):1785–93. doi: 10.1007/s00192-017-3481-6 (PMC5705742; doi:10.1007/s00192-017-3481-6)
Supplement: Supplementary file 1 — (DOCX 116 kb) [file 192_2017_3481_MOESM1_ESM.docx]

**Supplemental Material 1: Systematic Literature Review**

**Identification**

**Screening**

**Eligibility**

**Included**

**Identification**

**Screening**

**Eligibility**

**Included**

**Identification**

**Screening**

**Eligibility**

**Included**

**Identification**

**Screening**

**Eligibility**

**Included**

Full-text articles excluded
(n = 145)

Records identified through database searching
(n = 585)

Additional records identified through other sources
(n = 11)

Records after duplicates removed
(n = 586)

Records screened
(n = 586)

Records excluded
(n = 402)

Full-text articles assessed for eligibility
(n = 184)

Studies included in qualitative synthesis
(n = 39)

**Identification**

**Screening**

**Eligibility**

**Included**

Inclusion criteria -

- Studies investigating lower urinary tract

Exclusion criteria -

- Basic science / pathophysiology, neurogenic, pediatric, treatment complications, pharmacology, duplicates, case reports/series, editorials/responses, not available, review or book chapters, UDS parameters

Full-length articles reviewed and used to inform selection of outcomes and measures for Standard Set

**Identification**

**Screening**

**Eligibility**

**Included**

**Identification**

**Screening**

**Eligibility**

**Included**

**Identification**

**Screening**

**Eligibility**

**Included**
